# Supplementary material for: Planning for sustainable cities by estimating building occupancy with mobile phones
Source: Nat Commun. 2019 Aug 19;10:3736. doi: 10.1038/s41467-019-11685-w (PMC6700148; doi:10.1038/s41467-019-11685-w)
Supplement: Supplementary file 1 — Supplementary Information [file 41467_2019_11685_MOESM1_ESM.pdf]

Planning for sustainable cities by estimating building occupancy with mobile phones

Barbour et al.

Supplementary Information

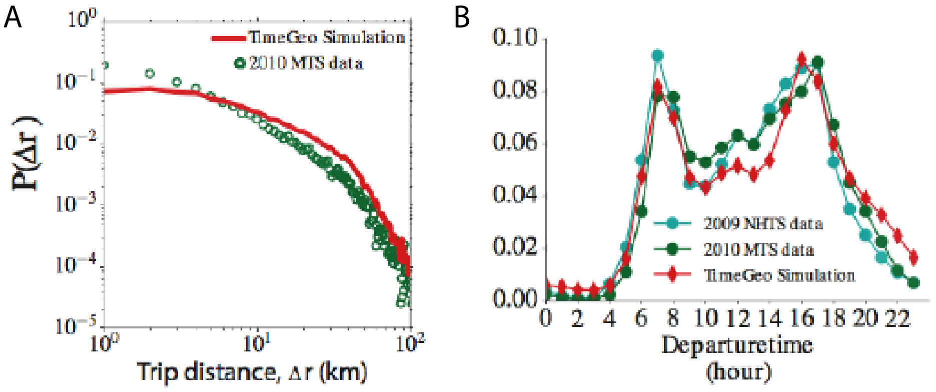

Supplementary Figure 1: (A) Comparing the trip distributions reported in the MTS with the TimeGeo simulation [1]. (B) Comparing the fraction of trip departures by time of the day between the TimeGeo, the 2010 MTS and the 2009 NHTS.

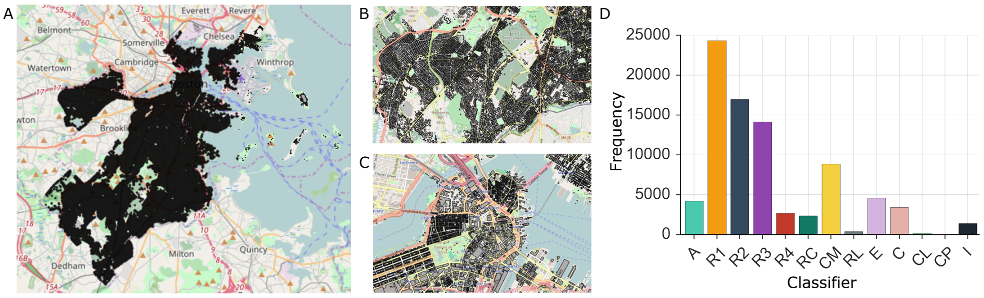

Supplementary Figure 2: The Building outlines available for the City of Boston. (A) Illustrating the extent of the City data. (B) Detailed building outlines in the Roslindale neighborhood in the southernmost extent of the city. (C) The Downtown Area which is home to Boston's financial district and has many high rise buildings. (D) Breakdown of the Boston Buildings dataset by classifier. Maps created using Google Maps, ©2019 Google.

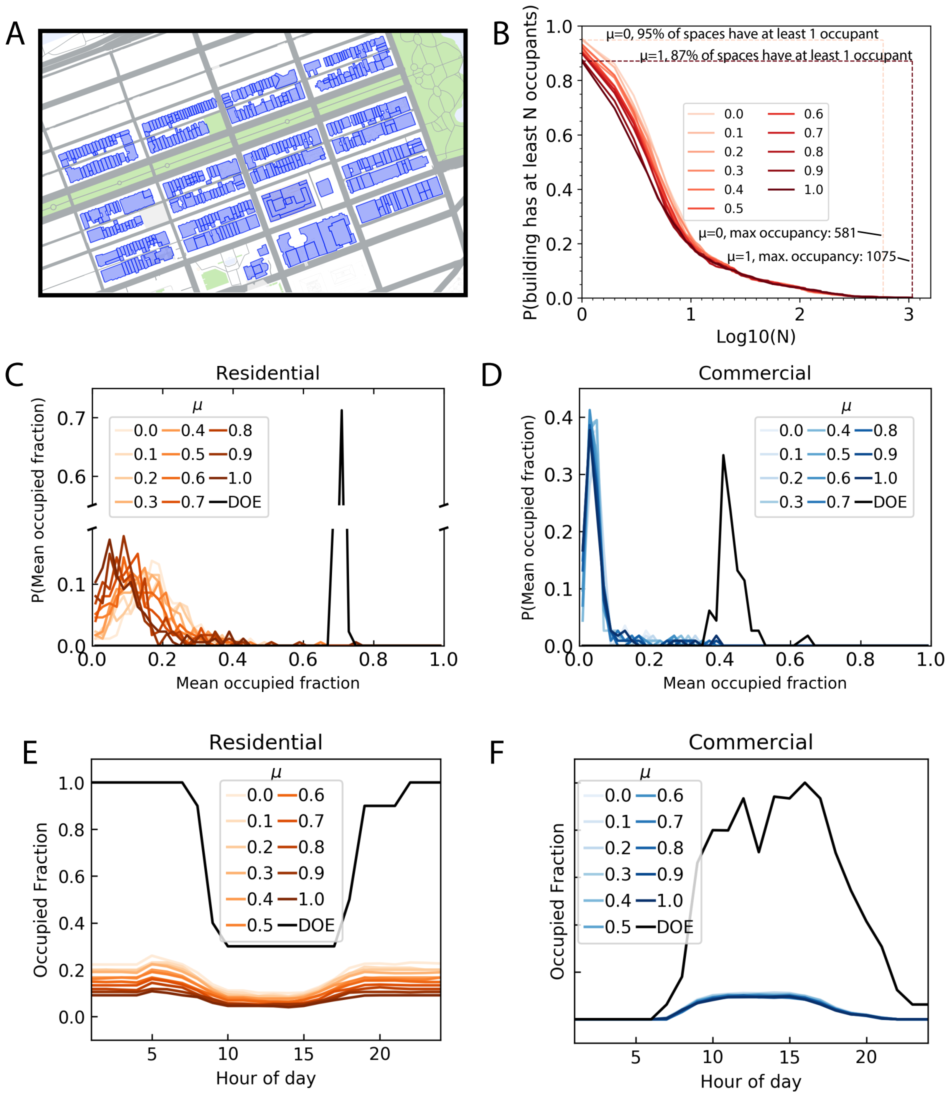

Supplementary Figure 3: Effect of the non-linear parameter μ. (A) The buildings in the back bay tract. (B) Probability of a space having at least $\log 10(N)$ occupants with different μ values. The number of unoccupied spaces and the maximum predicted building occupancy are also shown. (C) Probability distribution of the average occupied fraction of residential buildings. The black line illustrates the estimated occupied fraction assuming the DOE occupancy schedules. (D) Probability distribution of the average occupied fraction of commercial buildings. (E) Median daily occupancy fraction at different μ values for residential buildings. (F) Median daily occupancy fraction at different μ values for commercial buildings. The reference case value is also shown. Map created using Google Maps, ©2019 Google.

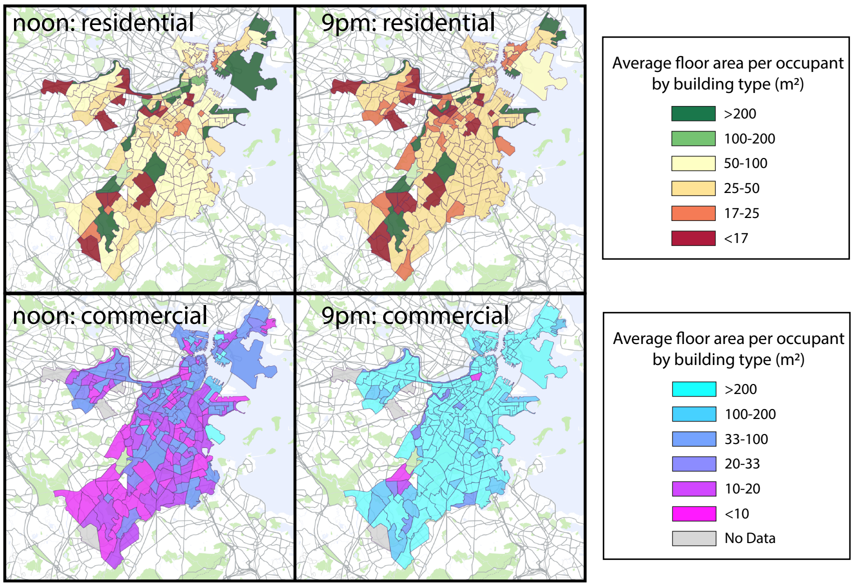

Supplementary Figure 4: Estimated floor space per occupant for residential and commercial buildings in different census tracts for the approximately 83,000 buildings in Boston. We note that residential occupant density is highly neighborhood dependent. Despite the fact that the absolute occupancy of commercial buildings is low in the predominantly residential neighborhoods in the bottom left portion of the map (see Figure 4 in the main text), the presence of several schools in these districts means the average occupant density for the commercial buildings in these areas is high. Furthermore, it is probable that our assumption that other stays occur only in commercial buildings is less satisfactory in these neighborhoods, since the ratio of residential-buildings-to-commercial-buildings is very high. Maps created using Google Maps, ©2019 Google.

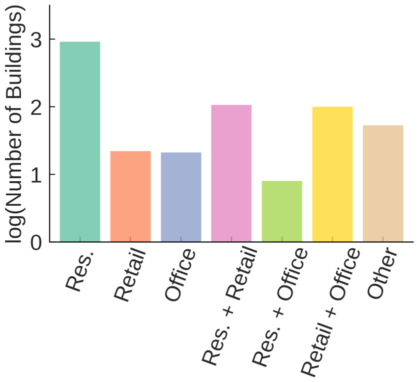

Supplementary Figure 5: Breakdown of the building types contained in the energy model

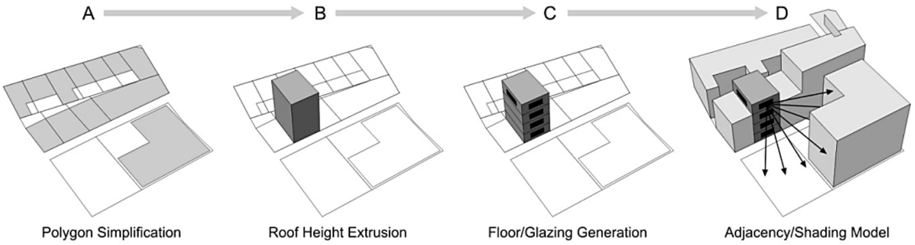

Supplementary Figure 6: Building 3D geometry generation in Rhino3d [2]

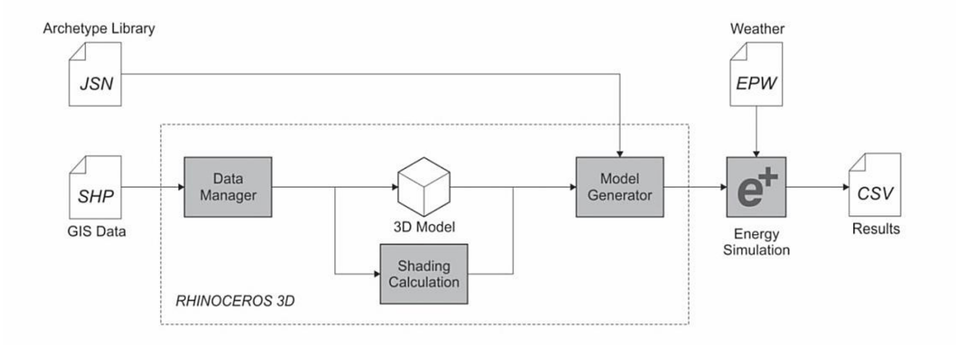

Supplementary Figure 7: The automated UBEM modeling workflow [2]

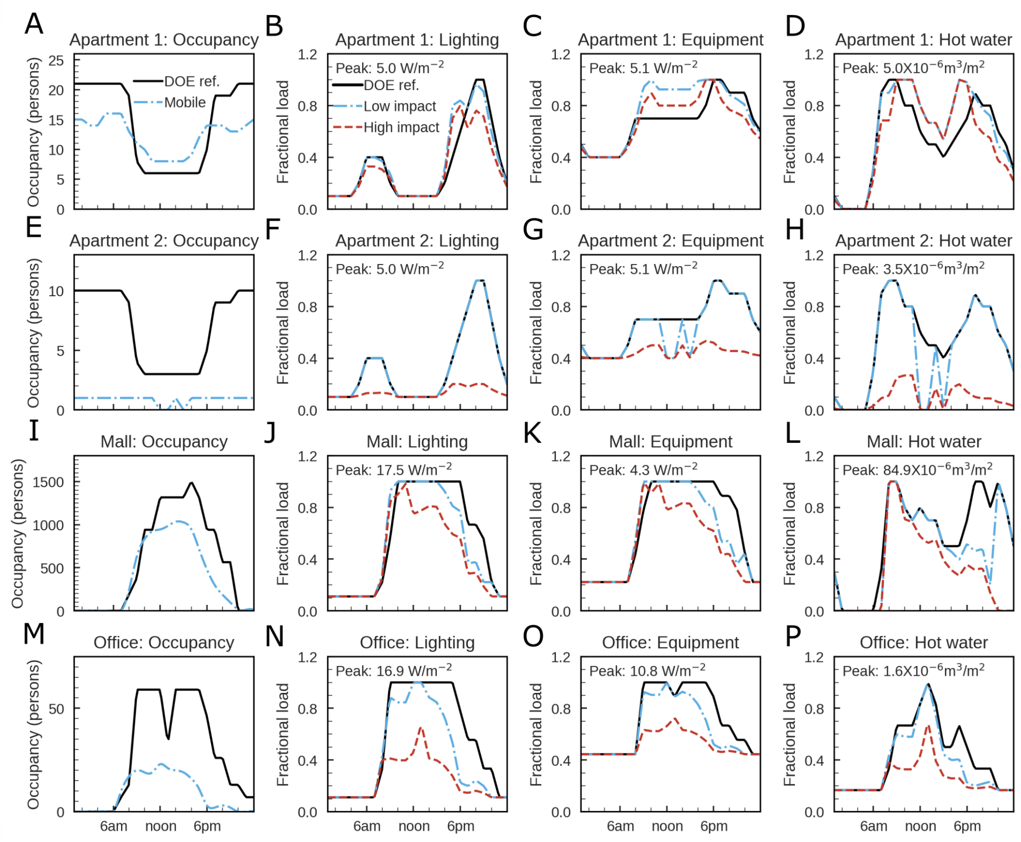

Supplementary Figure 8: Occupancy and occupancy-driven loads for example buildings. (A) Comparing the DOE reference buildings and mobile occupancy profiles for example apartment building 1. (B) The corresponding light schedule for each scenario. (C) The corresponding equipment schedules. (D) The corresponding hot-water schedules. (E) Comparing occupancy for example apartment building 2. (F) The corresponding light schedules. (G) The corresponding equipment schedule. (H) The corresponding hot-water schedules. (I) Comparing occupancy for an example retail building (Mall). (J) The corresponding light schedules. (K) The corresponding equipment schedule. (L) The corresponding hot-water schedules. (M) Comparing occupancy for an example office. (N) The corresponding light schedules. (O) The corresponding equipment schedule. (P) The corresponding hot-water schedules. Peak values to convert the fractional schedules to loads are also given.

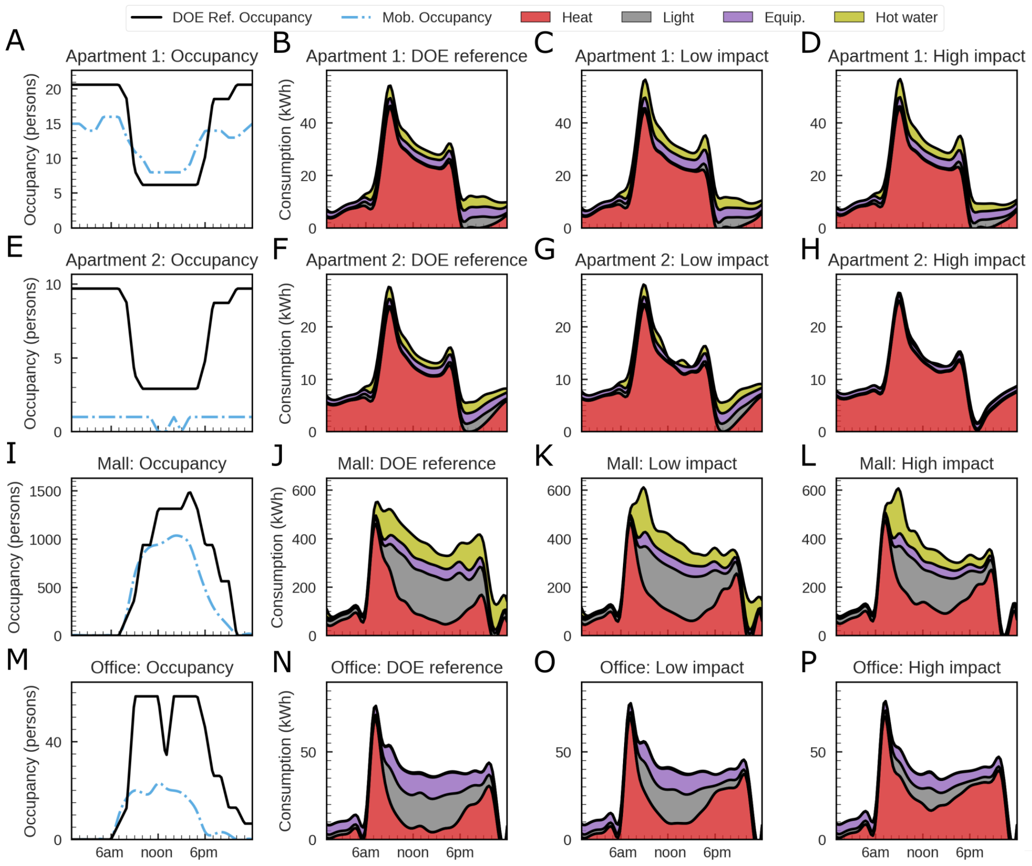

Supplementary Figure 9: Example building occupancies and energy consumption broken into end-use types in each scenario for the simulated winter day. (A) Occupancies for the DOE reference building model and our mobile-data method for an example apartment building. (B-D) Predicted energy consumption by end-use in the (B) DOE reference, (C) low-impact and (D) high-impact scenarios. (E-H) Apartment building 2. (I-L) Mall. (M-P) Office.

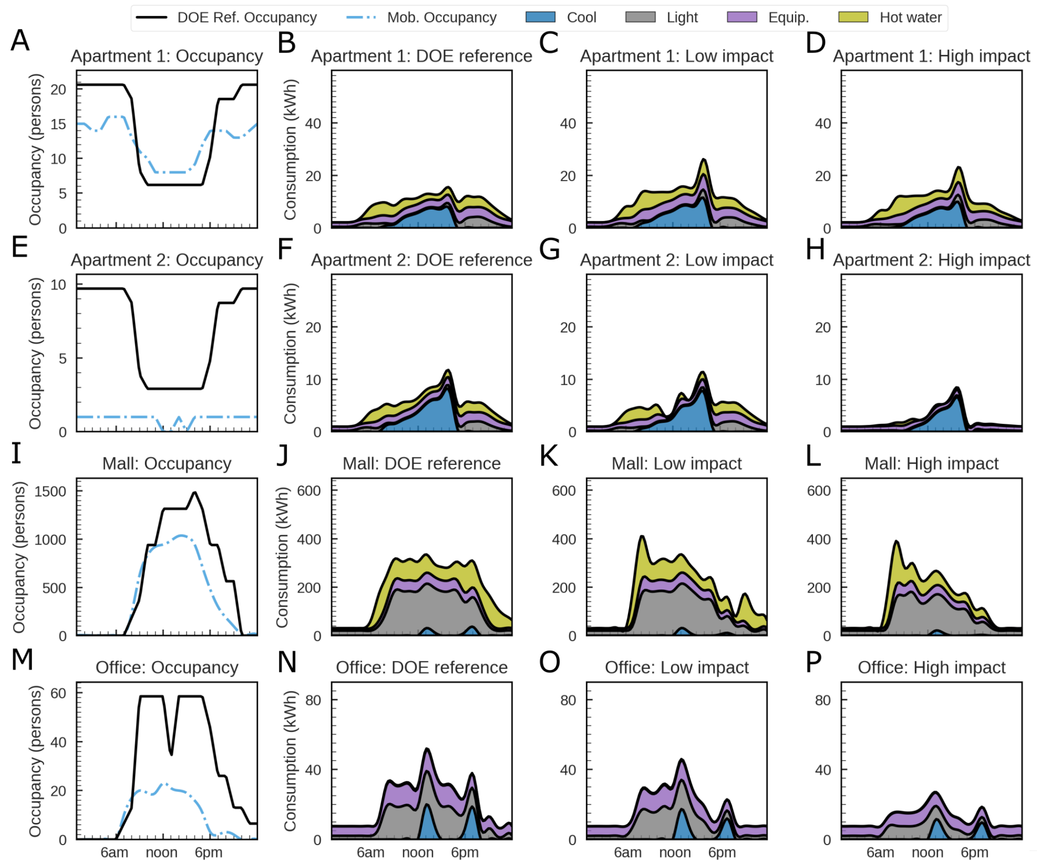

Supplementary Figure 10: Example building occupancies and energy consumption broken into end-use types in each scenario for the simulated summer day. (A-D) Apartment building 1. (E-H) Apartment building 2. (I-L) Mall. (M-P) Office.

Supplementary Table 1: The PCA values mapped to the functional use of buildings

Building type	PCA (m^2)
Athletic facility	4.6
College or academic	19.23
Convention centers	19
Fire/Police station	50
Garage	18.6
Hotel	8.06
Library	9.3
Medical lab	7.8
Museum	13.9
Office	19.23
Residential	40
Restaurant	1.86
Mall	6.2
Retail	7.1
School/daycare	3.23
Supermarket	12.82
Worship	1.4

Supplementary Table 2: The PCA mapped to the functional use of buildings

Building type	POI class
Athletic facility	gym, health club, bowling alley, spa, beauty salon, haircare
College or academic	university
Convention centers	movie theater, political, park, subway station, light rail station, transit station, train station, airport, bus station, night club
Fire/Police station	police station, fire station
Garage	car rental, car repair
Hotel	hotel, lodging
Library	library
Medical lab	dentist, doctor, physiotherapist, veterinary, hospital
Museum	art gallery, museum, aquarium
Office	bank, accountant, lawyer, general contractor, courthouse, car dealer, moving company, locksmith, plumber, electrician, city hall, local government office, embassy, funeral home, finance, insurance agency
Residential	—
Restaurant	cafe, bar, restaurant, food, meal, takeaway
Mall	shopping mall
Retail	store, book store, jewelry store, liquor store, electronics store, shoe store, clothing store, furniture store, pharmacy, home goods, florist, bakery, pet store, hardware store, bicycle store, movie rental, painter, travel agency, laundry, post office, real estate agency
School/daycare	school
Supermarket	grocery or supermarket, convenience store, department store
Worship	church, synagogue, place of worship, Hindu temple, mosque, stadium
None	ATM, street address, parking, premise, route, gas station, natural feature, neighborhood, RV park, Cemetery

Supplementary Table 3

cluster	Capacity. (other stays)	Probability ($\mu = 0$)	Probability ($\mu = 0.5$)
1	100	0.125	0.067
2	200	0.25	0.188
3	500	0.625	0.745

Supplementary Table 4: Aggregate results for all buildings.

		Low-Impact			High-Impact		
	DOE Ref.	$\mu = 0$	$\mu = 0.5$	$\mu = 1$	$\mu = 0$	$\mu = 0.5$	$\mu = 1$
daily total (MWh)	961.6	957.2	956.5	955.2	735.9	719.0	732.8
daily gas (MWh)	520.3	548.7	549.9	548.7	486.2	483.9	487.7
daily elec. (MWh)	441.4	408.6	406.6	406.5	249.7	235.2	245.0
winter gas (MWh)	797.7	873.0	877.2	874.1	875.1	880.8	878.1
winter peak elec. (MW)	28.1	24.4	24.4	24.4	12.0	11.2	11.6
summer peak elec. (MW)	43.2	37.2	36.7	36.7	23.3	21.8	22.7

Supplementary Table 5: Median percentage change in building energy use prediction for occupancy scenarios in with respect to the DOE reference scenario. A positive value denotes the median energy use is increased compared to the DOE reference scenario while a negative value implies the median energy use has decreased.

	Low-Impact			High-Impact		
	$\mu = 0$	$\mu = 0.5$	$\mu = 1$	$\mu = 0$	$\mu = 0.5$	$\mu = 1$
ΔE (total) (%)	-0.08	-0.03	-0.13	-15.99	-16.89	-17.86
ΔE (res. only) (%)	0.54	0.65	0.68	-14.21	-14.94	-15.88
ΔE (commercial) (%)	-4.19	-4.17	-4.02	-20.96	-20.98	-21.34

Supplementary Table 6: Median percentage change in energy use for residential buildings with upgraded insulation. All changes are calculated with respect to the equivalent simulation without the insulation upgrade. A positive value denotes a decrease in median energy use with the insulation upgrade while a negative value denotes an increase.

		Low-Impact			High-Impact		
	Ref	$\mu = 0$	$\mu = 0.5$	$\mu = 1$	$\mu = 0$	$\mu = 0.5$	$\mu = 1$
ΔE (total) (%)	2.32	2.41	2.42	2.43	2.92	2.92	3.01
ΔE (gas) (%)	3.79	3.76	3.97	3.80	4.23	4.26	4.29
ΔE (elec.) (%)	-0.13	-0.07	-0.07	-0.07	0.05	0.06	0.08

Supplementary Table 7: Median percentage change in energy use for commercial buildings with upgraded equipment. All changes are calculated with respect to the equivalent simulation without the equipment upgrade. A positive value denotes a decrease in median energy use with the equipment upgrade while a negative value denotes an increase.

		Low-Impact			High-Impact		
	Ref	$\mu = 0$	$\mu = 0.5$	$\mu = 1$	$\mu = 0$	$\mu = 0.5$	$\mu = 1$
ΔE (total) (%)	1.18	0.93	0.91	0.92	0.71	0.71	0.71
ΔE (gas) (%)	-2.44	-1.93	-1.78	-1.90	1.28	-1.24	-1.26
ΔE (elec.) (%)	2.88	3.19	3.12	3.20	4.37	4.31	4.43

Supplementary Note 1 Validation of the individual mobility model

Current mainstream urban mobility models require socio-demographic information from expensive manual surveys, which have small sample sizes and a low update frequency. TimeGeo [1] is an individual mobility modeling framework, generated from ubiquitous, passive, and sparse digital mobile phone traces. The model is able to generate individual trajectories at high spatial and temporal resolution. In this study, we simulate the mobility trajectories in Metro Boston at the metropolitan level. Supplementary Figure 1 illustrates that our model is consistent with the results of the 2009 National Household Travel Survey (NHTS) and the 2010-2011 Massachusetts Travel Survey (MTS). We also note that the underlying data and expansion to the city level is consistent with observed urban congestion [3].

Supplementary Note 2 Boston Buildings data

The Boston building dataset is provided freely by the city of Boston for research purposes, published by the Boston Department of Innovation and Technology in January 2012 [4]. The data entry for each building contains a variety of attributes including a unique id, a building type (within the city's designated tax classification scheme), the building geometry outline (a set of latitude/longitude pairs), the building's centroid point (latitude/longitude), the building height and the street address. There are a total of $82,542$ buildings in the dataset. Supplementary Figures 2A-C show the geographic extent of the data. The city's tax classification scheme includes apartments (A), condominiums (CM), residential land (RL), single(R1), double(R2), triple(R3) family dwellings, 4-6 apartment units (R4), commercial (C), exempt (E), industrial (I), 121-A property (EA) — a special tax class for urban redevelopment), commercial parking (CP) and multi-purpose residential (RC). The number of buildings by class is shown in Supplementary Figure 2D.

As mentioned in the main text, the tax classification scheme alone contains insufficient levels of information for attributing certain building properties which are important for the analysis, including building opening hours or Per Capita Areas (PCAs) for occupants based on building types. This is primarily because the commercial class designates a wide range of uses — for example including shops (which are typically open during the daytime hours) and restaurants (which are typically open later and can contain a much higher density of people). However, the tax classification scheme is useful for forming a broad segmentation of buildings into residential, commercial, industrial and mixed-use residential buildings. As described in the main text we stipulate that home stays can only be assigned to the individual's specific residence, work stays can be assigned to any building that isn't residential and other stays can be assigned to commercial buildings only. We split the buildings into these broad groups as outlined:

- **Residential:** A, CM, RL, R1, R2, R3, R4

- **Commercial:** C, E, EA
- **Industrial:** I
- **Mixed:** RC

We neglect the one building designated as commercial parking.

2.1 Mixed-use residential buildings

The RC building class has both a residential and a commercial component. By manually inspecting examples of the mixed use RC class, we find that in Boston this class typically exists as a row of single floor shops under residential space. Therefore, in our analysis we opt to split these buildings into the two constituent components — the residential part and the commercial part. We assume that if the building has more than one floor, the commercial part occupies only the first floor and the remaining floors are assumed to be residential space. If the building has only one floor, then the floor area is split equally between the residential and commercial components.

Supplementary Note 3 Building types to Per Capita Areas (PCAs)

Both in the estimation of occupancy based on cellphone data and in the later urban energy analysis of the case study district, it is necessary to estimate the maximum occupant capacity for each building. Since such information is not available, we estimate it from the gross floor area of each building, based on a typical Per Capita Areas (PCA) in occupants-per-square-metre for the building's functional use. For the case study area of Back Bay, we chose a total of 17 representative functional use types (see Supplementary Table 1), based on the use-type classification applied in the US Commercial Building Energy Consumption Survey (CBECS) [5] and the general land use classification by the City of Boston. Supplementary Table 1 shows the PCAs that we use in this study for each building functional use.

PCA values are difficult to find since they can vary largely for buildings of the same type. For that reason, in engineering and design they are often defined based on related limit requirements such as minimum ventilation rates or fire evacuation maximum occupancy. While useful for design, these limit values are commonly characterized by room type and not for complete buildings, and can overestimate the real occupant density in the building. In this study, the PCA values in Supplementary Table 1 are defined using two main sources. For those building types with a corresponding use in the DOE Reference Buildings dataset [6], PCAs use peak space occupancies as defined in the DOE reference models. These values were in turn mainly characterized according to maximum occupancy rates for ventilation as defined in the standard ASHRAE 62.1-2004 [7]. For those building types with no reference in the dataset (*i.e.* Athletic, FirePolice, Garage, Library, Museum and Worship), PCAs were chosen based on maximum Occupancy Load Factors for fire evacuation as defined by the

National Fire Protection Agency's NFPA 101-Fire Safety Code [8], adopted in the City of Boston's building codes. Finally, for the special case of the Hynes Convention Center, a unique structure within the study area, the average PCA was obtained based on the building capacities reported by the managing institution. These PCAs are used to estimate building capacity dependent on its classification within the set of types shown under the Building Type column. To obtain the capacity we divide the gross floor area by the PCA.

Supplementary Note 4 From POIs to buildings types

The Google Places API Web Service is a service that provides information about places contained in Google Maps. These Points Of Interest (POIs) are defined within the API as establishments, geographic locations, or prominent points of interest and can be accessed using HTTP requests. We use these POIs to obtain information regarding the functional uses of the non-residential buildings in our dataset. We use a two-stage querying process for returning POI details.

In the first stage, in each tract we find the set of non-residential buildings and query for POIs located around each non-residential building centroid within a designated search radius. This search radius in which we scan for POIs around each building centroid is important as it influences the type of results that we obtain from the query. For example, if the query radius is hundreds of kilometers, the names of towns and cities are returned while if the radius smaller we obtain richer local information. After manual testing and given typical maximum building sizes we select a radius of 50m for the queries. For each building centroid queried, a list of the unique IDs for each POI within the 50m search radius is returned. In each census tract we then collect the set of unique POI IDs near all the non-residential buildings in the tract.

In the second stage, we query each individual POI ID, returning the details available for each POI. This information can include many different fields, including place name, location, POI category, address, hours of operation, ratings, reviews *etc.*, however not every POI includes information for all of these fields. For example, reviews are not always available for restaurants and hours of operation are irrelevant for street names. Once we have the available information for all the POIs within each census tract, we use the POI location (latitude and longitude) to determine whether or not it is inside one of the non-residential buildings in that tract, forming a list of contained POIs for every non-residential building. This yields the set of POIs that lie within each non-residential building and each POI has a designated class. We then use the mapping outlined in Supplementary Table 2 to map the POI class to a building type in Supplementary Table 1, which can then be used to determine a PCA. The different entries in the POI class column of Supplementary Table 2 reflect the set of classes of POI that were located in the non-residential buildings in our dataset.

In the simplest case, a non-residential building contains one POI with information regarding class and opening hours. However, while some non-residential buildings contain

a single POI, others contain multiple POIs. If there is a single POI within the building, the building is assumed to have the functional use corresponding to that POI and the PCA is chosen accordingly from Supplementary Table 1. If there are multiple POIs within a building and they correspond to multiple possibilities of functional use and PCAs, the highest value PCA is assigned for the whole building. For opening hours, if there is one contained POI with information regarding opening hours then those opening hours are assumed to be valid for the whole building. If there is more than one POI contained with opening hours, then the building is assigned the opening hours corresponding to the superset of the opening hours for all contained POIs.

Supplementary Note 5 Effect of the non-linear parameter μ

The stay assignment process for assigning an individual stay to a building can be split into two steps.

1. Probabilistically assign each stay to a cluster of buildings.
2. Within this cluster, probabilistically assign the stay to an individual building.

Equation S1 for the first step states:

$$P(i) = \frac{\left[\frac{C_i}{\sum_{i=1}^{N} C_i}\right]^{1+\mu}}{\sum_{i=1}^{N}\left[\frac{C_i}{\sum_{i=1}^{N} C_i}\right]^{1+\mu}} \tag{S1}$$

For a given cluster i with a nominal capacity C_i, $P(i)$ is the probability of that cluster being assigned a particular stay. The total capacity of the cluster i is the sum of the capacity of the M_i buildings contained in cluster i. Therefore, $C_i = \sum_{j=1}^{M_i} C_{ij}$, where the buildings are indexed by j, and $C_{ij} = \alpha_{ij} A_{ij}$. α_{ij} and A_{ij} are the PCA and total floor area of building j in cluster i, respectively. The parameter μ introduces the non-linearity and the degree of preferential attraction to regions with a high-capacity for stays of that type.

Once a cluster i has been selected, then within that cluster we assign the stay to a building j with probability $P(j|i)$ proportional to it's relative within-cluster capacity, as described by Equation 3.

$$P(j|i) = \frac{C_{ij}}{\sum_{j=1}^{M_i} C_{ij}} = \frac{C_{ij}}{C_i} \tag{S2}$$

Our rationale for adopting this approach is as follows:

- The rich-get-richer effect has been observed in many forms of empirical data relating to society, human-behavior and networks [9]. Importantly, it has also been observed in population and city-size growth, as well as many other areas [10]. Therefore we

postulate that higher capacity regions will attract more people and thus stays are preferentially attracted to neighborhoods with larger capacities for that type of stay.

- We do not anticipate diminishing returns, that is we do not expect that an area with increased capacity will attract fewer stays per unit capacity. Therefore we expect that $\mu \geq 0$.
- Agglomeration of shops and industries is observed due to clustering of economic activities [11] — for example the formation of shopping or restaurant districts.
- Within each cluster, we assume that each building has the same opportunity to attract stays, and therefore, the non-linear effect is only present at the city-block level.

The effect is described in the following example. Consider that we have a stay of type other which can be mapped to commercial buildings and the buildings in the stay tract are clustered into three blocks. Cluster 1 has a capacity of 100 other stays (a couple of small shops), cluster 2 has a capacity of 200 and cluster 3 has a capacity of 500 (a shopping mall). The probability of the stay being assigned to each cluster is shown in Supplementary Table 1. Upon inspection of Equation S1, we can see that when $\mu = 0$, we have that each cluster of buildings attracts stays proportional to its capacity. That is to say that, we expect that the number of stays per-unit-capacity is the same for all buildings in all locations (within a tract). Conversely, when $0 \leq \mu \leq 1$, we find that higher capacity clusters attract disproportionately more stays, emphasizing the rich-get-richer effect. We do not anticipate that $\mu > 1$, using intuition from network theory, wherein $\mu > 1$ represents a winner-takes-all regime.

For an example tract in the Back Bay neighborhood (Supplementary Figure 3A), Supplementary Figure 3B shows the probability that a given building has a maximum occupancy of more than N occupants, illustrating the effect of changing the non-linear parameter μ. As μ increases, people are shifted from lower occupancy spaces to higher occupancy spaces — so with increasing μ the number of unoccupied spaces increases and there are fewer low-occupancy spaces predicted. Correspondingly, the probability of spaces with very high occupancy levels increases with μ. We find that as μ approaches 1, the maximum occupancy in certain spaces becomes unreasonably large. In particular, we find that when $\mu = 1$ the space with the highest predicted occupancy is a large residential building where we expect that the maximum occupancy would not exceed $1,000$ people. The predicted occupancy for this space doubles as μ increased from 0 to 1.

5.1 Comparison to DOE reference occupancy

Supplementary Figure 3C shows the probability distribution of the average daily occupied fraction of all residential buildings in the tract compared to the maximum DOE stated occupancy and Supplementary Figure 3D shows the equivalent for non-residential buildings. The average daily occupied fraction is expressed as follows:

$$\hat{O}^{\text{mob}} = \frac{\sum_{h=1}^{24} O^{\text{mob}}(h)/\text{MAX}\{O^{\text{DRB}}\}}{24} \tag{S3}$$

For comparison the average daily occupied fraction for the DOE reference buildings occupancy is also shown. The corresponding occupied fraction in the DOE reference model is $\hat{O}^{\text{DRB}} = \frac{\sum_{h=1}^{24} O^{\text{DRB}}(h)/\text{MAX}\{O^{\text{DRB}}\}}{24}$. Supplementary Figure 3E shows the median occupied fraction throughout the day for all residential buildings compared to the DOE occupancy schedules and Supplementary Figure 3F shows the equivalent for commercial buildings. we can see that the effect of changing the parameter μ is small compared with the differences between the DOE Reference Buildings occupancy and the mobile-inferred occupancy.

Supplementary Note 6 The Urban Building Energy Model

We construct an Urban Building Energy Model (UBEM) for the 1,266 buildings in the Back-Bay area of Boston, where we have sufficient detailed information regarding the building geometries and uses per floor. The extent of these buildings is illustrated by Figure 1 in the main text and Supplementary Figure 5 shows these buildings broken down by type. While on the individual building level the procedures for generating Building Energy Models (BEMs) normally involve hiring expert energy modelers with extensive knowledge of architectural engineering, in this study we rely on a streamlined and automated multi-tool workflow developed for the sole purpose of UBEM generation. The applied workflow uses the dynamic thermal simulation engine EnergyPlus [12, 13] to calculate the energy demands of every building in the model. This workflow, built around the UMI framework [14] and which has also previously been used to create a less detailed citywide model for Boston [2], relies on three main data inputs: Hourly weather data, building geometry, and building type average characteristics (commonly assigned through archetypes). These inputs are characterized as follows:

- **Weather data.** Hourly level data for measured environmental variables such as solar radiation, dry bulb temperature, relative humidity, and wind speed and direction need to be characterized in order to run an energy simulation. Average representations of these variables for a given location are usually stored using the Typical Meteorological year (TMY) file format [15]. TMY data is provided for most major cities in the US by the Department of Energy using the EPW file format [16]. For this study, the TMY3 (3rd generation) data for Boston Logan Airport was used, which is representative of the average weather for a station located approximately 3 miles away from the Back Bay area.
- **Input building geometry.** UBEM models require 3D representations of a building's massing, the glazed areas in its façade, and its neighboring structures for shading purposes. To create these models, the GIS datasets for building footprint polygons,

as gathered by the City of Boston for Fiscal Year 2014, were first simplified to avoid unnecessary geometric detail (models with a large number of surfaces which thermal simulation programs are either unable to resolve or which unduly prolong simulation times). We used the standard polygon simplification method available in the GIS ArcMap software [17] and a subsequent time-consuming manual quality control step. Building heights and number of floors were obtained from both the City of Boston GIS and Property Tax Assessment databases for the same year. In the absence of building by building window to wall ratio (WWR) information, glazing areas by facade were assigned according the typical values defined in the DOE Reference Building dataset [6]. Once processed, the simplified building footprints, heights and WWR values available were combined in a single GIS dataset.

- **Building archetype characteristics.** In addition to purely geometric data about building shape, dynamic thermal simulation models require the definition of many non-geometric inputs, including thermal properties of the envelope and glazing, efficiency data for building systems, and hourly values for the occupant related loads for lighting and equipment use explored in this paper. Since this information is not available at the urban scale, UBEMs typically rely on average descriptions of buildings of a particular use type and period of construction, commonly referred to as archetypes [18]. As mentioned in the main text, the DOE commercial reference buildings dataset provides the standard set of templates for the US building stock for the EnergyPlus simulation engine, and constitutes a common accepted source for defining building archetypes. It contains definitions for every model input for 16 building use-types across periods of construction and climate zones in the US.

  Since no Boston specific building data source was available at the time of this study, and to guarantee its reproducibility, we have chosen to only model the energy use of buildings falling within the classes available in the DOE dataset. Hence, for the Back Bay case study, archetypes were defined for the 9 of 17 building use types in Table 1 included in the DOE Reference Building's dataset, for 4 periods of construction: Built prior to 1950, 1950 to 1979, 1980 to 1999, and 2000 to present, and for Climate Zone 5A. All data inputs by archetype were stored in a library file in JSON format. In order to assign archetypes to the buildings in Back Bay, we used their use type and year of construction as reported in the City of Boston City of Boston GIS and Property Tax Assessment databases. For those buildings marked as mixed-use, Google POI information was used to assign different use archetypes by floor when necessary, resulting in mixed categories such as Residential+Retail or Office+Retail.

Starting with the GIS dataset defined before, 3D models for building floor volumes, facade, glazing, and context shading are generated within the CAD environment Rhino 3D [19] and its parametric modeling tool Grasshopper [19] using a set of custom C# applications. Building footprint polygons were extruded to the documented height, and divided into floors

(see Supplementary Figure 6). Once data inputs for the climate, massing, and archetypes are selected, each building considered in the UBEM model is digitally reconstructed and its energy use calculated using the EnergyPlus thermal simulation engine. The engine will calculate all heat and mass flows in the building, according to climate, its constructions, and its internal usage patterns, and estimate the resulting heating and cooling needs. The previously referred UMI based workflow, takes GIS geometry, an archetype library file, and an EPW file as inputs and processes each building before sending it to EnergyPlus as depicted in Supplementary Figure 7. Simulation parameters for each archetype were stored and implemented in a JSON library file format [20]. Finally, archetype data was associated with each building within Grasshopper, and used to generate individual EnergyPlus input files (IDFs) using the UMI tool framework. After running EnergyPlus simulations of each produced IDF file, results by building were obtained in text file format for five energy end uses: Heating needs, cooling needs, lighting usage, equipment usage and hot water consumption. These results were further aggregated into electricity and gas hourly, monthly and annual needs per building.

Supplementary Note 7 Occupancy Scenarios

The differing building occupancies predicted by our method using mobile phone data as compared to the DOE reference buildings occupancy schedules suggest that the building load patterns should also differ significantly. For example, at times when a building's predicted occupancy using the DOE reference buildings is low and the occupancy predicted using mobile phone data is high, the energy consumption profile of the building associated with the mobile phone inferred occupancy should be higher.

Although EnergyPlus takes occupancy as a simulation input, it makes no explicit prediction regarding active occupant behavior — the only direct effects of changing the input for occupancy are related to passive occupant behavior, *i.e.* changing occupancy changes the amount of body-heat produced by the occupants and the internal CO_2 production (which requires ventilation) in a building, but does not change the building's predicted plug loads (from computers, kitchen equipment, *etc.*). Instead, EnergyPlus also takes operational schedules for the occupant driven loads, namely equipment, lighting and hot water loads, as inputs. Therefore, to achieve realistic simulations, when the input for occupancy is changed the inputs for the operational schedules of the different active-occupancy-driven loads must also be changed.

The two scenarios for the impact of occupancy on the load schedules of buildings that we develop are described by Equations 2 (low-impact scenario) and 3 (high-impact scenario) in the main text. These relate the hourly values of the predicted mobile load L^{mob} and the DOE reference building loads L^{DRB}, dependent on the mobile and reference building occupancies, O^{mob} and O^{DRB} respectively.

The low-impact scenario represents the case where the standard predicted loads are representative for the relative occupancy of the building (*i.e.* if the DOE reference building and mobile fractional occupancies of the building are the same at a particular hour then the predicted loads will be the same) and the high-impact scenario represents the case where the template loads are representative for the absolute occupancy of the building (*i.e.* only if the DOE reference building and mobile occupancy schedules predict the same number of people will the predicted loads be equal). Using building science intuition, the low-impact scenario is expected to be a good representation of buildings where occupancy typically drives when building appliances are ON or OFF, but the exact number of active occupants has a small impact on the actual load levels. For example, this may be the case in an open-plan office or retail building where lights or fans are ON as soon as there is one person in the space, but the level of consumption doesn't change by a large amount with the presence of additional occupants. The high-impact scenario is expected to work best in compartmentalized building types, such as apartment buildings, where the number of active appliances depends strongly on the absolute number of active occupants, *i.e.* we expect a bedroom light will be on in each bedroom where there is an active occupant and there are low numbers of occupants per bedroom.

However, upon close inspection we see that Equation 2 for the low-impact scenario leads to unlikely results if the number of occupants predicted by the mobile occupancy schedule is much lower than that for the DOE reference building occupancy, but simultaneously the mobile occupancy represents a higher percentage of the maximum mobile occupancy. In this specific case, the loads can be predicted (under the low-impact scenario) to be significantly higher than the predicted load for the DOE reference building, even though the absolute number of occupants is much smaller.

Therefore, we enforce the following three stipulations:

1. In the low-impact scenario we stipulate that if the predicted mobile load is higher, it can only be higher than the DOE reference building load by a factor of $O^{\text{mob}}(h)/O^{\text{DRB}}(h)$.

2. In both scenarios, if the maximum DOE reference building occupancy is higher than the mobile occupancy at hour h, $\text{MAX}(O^{\text{DRB}}) >= (O^{\text{mob}})$, then the maximum predicted load must be less than or equal to maximum value in the reference buildings scenario, *i.e.* $L^{\text{mob}}(h) <= \text{MAX}(L^{\text{DRB}})$.

3. In both scenarios, if the maximum mobile occupancy is higher than the maximum DOE reference building occupancy, *i.e.* $\text{MAX}(O^{\text{mob}}) > \text{MAX}(O^{\text{DRB}})$, then the predicted load at any time can only be higher than the standard load by a factor of $\text{MAX}(O^{\text{mob}})/\text{MAX}(O^{\text{DRB}})$.

Supplementary Figure 8 illustrates the occupancy-driven load schedules (lighting, equipment and hot-water) and the occupancy of four example buildings. These include two different Apartment buildings, a Mall (retail) building, and a medium Office.

Supplementary Note 8 Additional Energy Use results

Running EnergyPlus simulations for each building yields hourly predictions of building energy consumption broken into five different categories for each simulated building zone. The five different load categories are:

1. Zone Lights Electric Energy
2. Zone Ideal Loads Supply Air Total Cooling Energy
3. Zone Ideal Loads Supply Air Total Heating Energy
4. Zone Water Use Equipment Heating Energy
5. Zone Electric Equipment Electric Energy

We assume that total electric usage is composed of the energy consumption for lighting loads, cooling loads and electric equipment loads and that total gas consumption is composed of energy use for heating and energy use for hot water. We recognize that there is a significant variation in the correspondence of these energy vectors (*i.e.* electricity can be used for space-heating or natural gas can be used to power cooling systems) to different consumption end-uses on a building-by-building basis, however this segmentation is reflective of the typical status quo our modeled region.

We run EnergyPlus simulations for all buildings with occupancy as prescribed by $\mu = 0$, $\mu = 0.5$ and $\mu = 1$ (see Supplementary Section 5 for further information regarding the effect of the parameter μ). These simulations yield a range of energy predictions which correspond to the uncertainty in the occupancy distribution in our model. Figures 5A-5D in the main text illustrate the most important details of these results visually, showing the distributions of daily Energy Use Index (the daily energy use per unit floor area) for all buildings in each scenario (DOE reference, low-impact and high-impact) for each of the occupancy distributions ($\mu = 0$, $\mu = 0.5$ and $\mu = 1$). Total predicted hourly electricity loads (aggregated for all buildings) for the simulated typical-winter and typical-summer days are also shown. The profile of electricity consumption is particularly important for system operators to understand and predict, as they need to be able to deliver electricity as and when it is needed. This requires both having sufficient generation capacity to provide the maximum power but also having sufficient flexibility to cope with sudden changes in demand. Variations in hourly gas demand are less consequential, as there is a large amount of inherent storage capability within the gas network. However, accurate estimates of the total gas usage on a day-to-day basis are crucial for suppliers to ensure that they always store sufficient gas to cover extended cold periods in winter.

Supplementary Table 4 provides further details about the aggregate energy results for each set of simulations. The median building-level changes implied by the low-impact and high-impact scenarios for all occupancy distributions are shown in Supplementary Table 5.

Supplementary Table 4 demonstrates that the differences in the aggregate results between the different occupancy distributions are small compared to the differences between the different occupancy scenarios. However, comparing the median building level changes, as shown in Supplementary Table 5, we see that in the high-impact scenario there is a clear trend for larger energy use reductions with larger μ values. This is explained as the larger μ value tends to result in more highly populated buildings and thus in general lowers the median building occupancy (see Supplementary Section 5 and Supplementary Figure **??**). Therefore, the building median energy use is noticeably decreased with increasing μ in the high-impact scenario.

8.1 Demonstrating the occupancy scenarios in individual buildings

Illustrating the results for different individual example buildings and the effect of occupancy in each scenario, Supplementary Figure 9 shows the results for the winter day simulation for the four different example buildings (two apartments, a mall and an office) previously considered in Supplementary Figure 8. Correspondingly, Supplementary Figure 10 shows the results for the summer day simulation for the same four buildings.

We can see that, compared to the DOE reference scenario, the low-impact scenario for Apartment 1 predicts that the equipment, hot water and lighting loads are increased during the day. This is due to the larger number of occupants present in the building during the day as predicted by the mobile occupancy compared with the DOE reference occupancy. Both the low-impact and high-impact of occupancy scenarios predict similar results for this building since the mobile occupancy is similar to the DOE reference model. Conversely, for Apartment 2 the equipment, hot water and lighting loads are predicted drastically reduced in the high-impact scenario, since there is a maximum occupancy of a single person for the mobile occupancy compared to the maximum DOE occupancy of 10 occupants. We can also see that for both Apartment 2 and the Office, the heating loads are much higher during winter due to the much lower mobile occupancy.

Supplementary Note 9 Building energy efficiency measures

As discussed in the introduction to the main text, one particularly compelling use for UBEMs is to understand potential outcomes resulting from energy policies which encourage the adoption of certain energy efficiency measures. To study this and the effect of occupancy patterns, we devise two energy efficiency scenarios which are intended to represent the outcomes of different potential energy policies. The two scenarios are described as follows:

1. **Insulation upgrade**. We improve the wall and roof insulation in all the simulated residential spaces. The different model archetypes described in Supplementary Section 7 have different wall constructions dependent on the build period (prior to 1950, 1950-1979, 1980-1999, 2000-present). For the insulation upgrade we improve the wall and

roof insulation by 10% (i.e. we increase the R-value by 10%) in comparison to the 2000-present construction, and apply this level of insulation to all the simulated residential zones. This could represent the upper limit of the impact of a policy to subsidize insulation retrofits in any residences with insulation below this standard.There are 1040 buildings in our model which contain at least one residential zone.

2. **Equipment upgrade**. For commercial zones, including office zones, retail zones (including shopping Malls) and restaurants, we implement a blanket improvement of 10% in the equipment efficiency. This could represent the maximum impact of a policy of offering incentives for commercial spaces to upgrade to high efficiency appliances. There are 296 buildings in our model with at least one of these space types.

To manipulate the EnergyPlus input files (idf files) and implement the energy efficiency measures, we use Python and Eppy [21], a scripting package for EnergyPlus.

To implement the insulation upgrade, we filter through all the simulation input files for zones which are designated for residential use, and increase the wall and roof insulation thickness by 10% in comparison to the 2010 standard.

For the equipment upgrade, we filter through all the building idf files for any office, retail, mall or restaurant zones and reduce the peak value for equipment electricity usage per square meter by 10%.

Supplementary Table 6 illustrates the median reduction in building energy use for the 1040 buildings for which we do the insulation upgrade. We again find that the differences between the occupancy distributions are significantly smaller than between the occupancy scenarios. Similarly, Supplementary Table 7 illustrates the median reduction in building energy use for the 296 buildings for which we do the equipment upgrade.

Supplementary References

[1] Jiang, S. *et al.* The timegeo modeling framework for urban mobility without travel surveys. *Proceedings of the National Academy of Sciences* **113**, E5370–E5378 (2016).

[2] Davila, C. C., Reinhart, C. F. & Bemis, J. L. Modeling boston: A workflow for the efficient generation and maintenance of urban building energy models from existing geospatial datasets. *Energy* **117**, 237–250 (2016).

[3] Çolak, S., Lima, A. & González, M. C. Understanding congested travel in urban areas. *Nature communications* **7**, 10793 (2016).

[4] City of Boston. City of Boston open data hub (2018). URL `https://data.boston.gov`.

[5] Energy Information Administration. Commercial Buildings Energy Consumption Survey (2012). URL `https://www.eia.gov/consumption/commercial/`.

[6] US Department of Energy. Commercial Reference Buildings (2018). URL https://energy.gov/eere/buildings/commercial-reference-buildings.

[7] Standard, A. Standard 62.1-2004,". *Ventilation for acceptable indoor air quality* (2004).

[8] National Fire Protection Association. *Life safety code handbook: with the complete text of the 2015 edition of NFPA 101, Life safety code* (2015).

[9] Barabási, A.-L. & Albert, R. Emergence of scaling in random networks. *science* **286**, 509–512 (1999).

[10] Perc, M. The matthew effect in empirical data. *Journal of The Royal Society Interface* **11**, 20140378 (2014).

[11] Fujita, M. & Thisse, J.-F. *Economics of agglomeration: cities, industrial location, and globalization* (Cambridge university press, 2013).

[12] Crawley, D. B. *et al.* Energyplus: creating a new-generation building energy simulation program. *Energy and buildings* **33**, 319–331 (2001).

[13] US-DOE Office of Energy Efficiency and Renewable Energy. EnergyPlus V8.4 [software] (2018). URL https://energyplus.net/weather.

[14] Reinhart, C., Dogan, T., Jakubiec, J. A., Rakha, T. & Sang, A. Umi-an urban simulation environment for building energy use, daylighting and walkability. In *13th Conference of International Building Performance Simulation Association, Chambery, France* (2013).

[15] Crawley, D. B., Hand, J. W. & Lawrie, L. K. Improving the weather information available to simulation programs. In *Proceedings of Building Simulation'99*, vol. 2, 529–536 (Citeseer, 1999).

[16] US Department of Energy. EnergyPlus weather data (2018). URL https://energyplus.net/weather.

[17] ESRI. ArcGIS suite (2018). URL https://desktop.arcgis.com/en/desktop.

[18] Reinhart, C. F. & Davila, C. C. Urban building energy modeling–a review of a nascent field. *Building and Environment* **97**, 196–202 (2016).

[19] Robert McNeel & Associates. Rhinoceros - NURBS modeling for windows V6 [software] (2018). URL https://www.rhino3d.com.

[20] Cerezo, C., Dogan, T. & Reinhart, C. Towards standardized building properties template files for early design energy model generation. In *Proceedings of the ASHRAE/IBPSA-USA building simulation conference*, 25–32 (2014).

[21] Santosh, P. Eppy 0.5.48 [software] (2018). URL https://pypi.org/project/eppy/.
